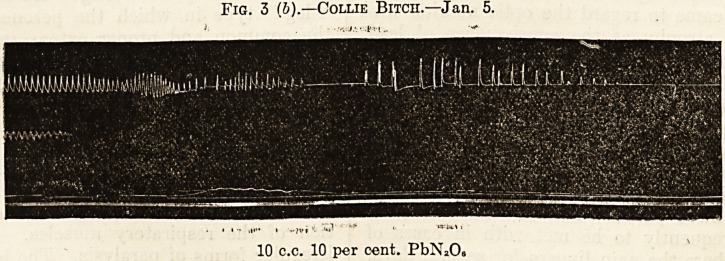# Some Unusual Features of Lead Poisoning

**Published:** 1909-05-29

**Authors:** Thomas Oliver

**Affiliations:** Physician to the Royal Victoria Infirmary, and Professor of Physiology in the College of Medicine, Newcastle-upon-Tyne


### Hospital Clinics.

### SOME UNUSUAL FEATURES OF LEAD POISONING.

By Sir THOMAS OLIVER, M.D., LL.D., D.Sc., F.R.C.P.; Physician to the Royal Victoria Infirmary, and Professor of Physiology in the College of Medicine, Newcastle-upon-Tyne.

(A Lecture Delivered at The Polyclinic, May 19, 1909.)

Although an old subject lead poisoning still offers many points for consideration. It may, therefore, not be a waste of time to-day if we discuss together some of the direct and indirect issues which plumbism raises. Notwithstanding all that the Home Office has accomplished and the saving of human life and suffering which its regulations have effected, there is yet an amount of industrial lead poisoning which is a source of anxiety to the legislature and a disappointment to employers. With the march of civilisation new industries in which lead is used are continually developing, new methods of manufacture are constantly being introduced whereby fresh sources of plumbism arise. At their inception these industries are usually carried on with care that no illness occurs. Later on, as the older workmen become replaced by less experienced hands, all at once, or gradually, symptoms of saturnine poisoning appear. The operation of the Workmen's Compensation Act has given a fresh impetus to the subject of lead poisoning, for additional responsibilities have been imposed upon members of the medical profession. With plumbism as with appendicitis, there is a tendency for the number of cases to increase as public and professional attention is directed to it. Even with lead workers it is scarcely proper to attribute all their ailments to lead. These may be due to other causes, such as deranged digestion, the results of alcohol and improper feeding. An open mind ought to be the attitude of every medical practitioner towards the subject of industrial lead poisoning, each case requiring careful consideration, for while it is unwise to regard all trifling symptoms in workmen as the consequences of their occupation, it is equally a misfortune to overlook plumbism when present. Within recent years, since the abolition of female labour in lead works, the cases of plumbism which have come under my care have been of a less acute character and have taken a longer time to recover. To some extent lead poisoning differs in different trades. While in the main one of daily dosage and rapidity of absorption, there also enter into its causation such circumstances as fatigue, nutrition, respiration by the mouth, indulgence in alcohol, and alternation of employment. Workers in some trades suffer mostly from gastro-intestinal disorders, while in others nervous, kidney, and cardio-vascular derangements predominate. To some physicians it is a debatable point as to whether there is any difference in plumbism produced rapidly and slowly. My own feeling is that the worst cases, generally speaking, are those where the poison has entered imperceptibly and been absorbed slowly over a long period in extremely minute quantities. I have said generally, so as to exclude acute saturnine encephalopathy, which is admittedly one of the most serious forms of lead poisoning. Under the other circumstances to which I have alluded the effects of lead are cumulative, the structural changes which are slowly produced are permanent, and under

Fig. 1.—Fox Terrier (Puppy—Male). (Ether.)

(*a*) (*b*) (*c*) (*d*)

5 c.c. Aq. Dest. | 5 c.c. 1 per cent. $PbN_2O_6$ | 5 c.c. 5 per cent. $PbN_2O_6$ | 5 c.c. 10 per cent. $PbN_2O_6$

Fig. 2.—Fox Terrier (Male). (Ether-Chloroform.)

5 c.c. 10 per cent. $PbN_2O_6$

treatment elimination is tardy. The excretion of lead is always slower than its absorption, hence the tendency for the poison to accumulate in the system. To the part played by leucocytes in the absorption, transference, retention, and elimination of lead we will later on refer.

The proneness of certain families and individuals to infectious diseases is common knowledge. A similar idiosyncrasy is observable in lead poisoning. Some persons are more readily brought under the influence of lead than others. Of young persons and especially females is this particularly true. It is because lead rapidly affects the blood-making organs of the body that anæmic subjects do not bear exposure to it. Still, this is only one factor. I have seen a young female lead worker die from acute saturnine encephalopathy after two months' work in a white lead factory, and, on the other hand, I have seen a strong, healthy joiner out of work suffer from agonising colic after three months casual labour in a red lead factory. Behind apparent health there are often idiosyncrasy and the predisposing effects of poverty to be dealt with. As an illustration of idiosyncrasy, I have seen a whole family of sons die from lead poisoning. We are familiar with this aspect of the subject, namely, diminished resistance to lead, but we do not hear so much of a resistance to it. This, like its opposite, is often a family trait. One of my infirmary patients, a man aged 45, commenced work in the smelting department of a lead works thirty years ago; for the last twenty-three years he has been in the red lead department. Until December of last year his work has never been interrupted by illness. His father is an old man of 80, who worked for forty-four years as a red lead maker, and never suffered. The patient has three brothers who have worked for several years in lead, and not one of them has suffered from plumbism. I have seen similar resistance to lead exhibited by animals. In going through a lead factory it is not uncommon to find one or two old workers who have never been ill, but the immunity is not absolute, for it only requires the development of an intercurrent malady such as influenza, the receipt of an injury, or a more than usual indulgence in alcohol, for severe symptoms of plumbism to arise. Under these circumstances the resistance of the individual becomes changed, or the constitution of his blood and fluids becomes altered, whereby lead long lying dormant in an insoluble form in the tissues is re-dissolved, circulates in the blood, and sets up lead poisoning. It is difficult to say how long lead can remain in the body without causing symptoms. Two cases bearing upon this point have recently come under my notice. (1) A married woman, aged 36, was admitted into the infirmary suffering from severe headache, paralysis of the muscles of one eye-ball, and defective eyesight. She was the mother of seven children, six of whom were alive and healthy. There was a history of three miscarriages—probably the result of her occupation as a white lead worker—before her marriage. I recognised this woman as an old patient whom I had treated seventeen years previously for encephalopathy, followed by blindness, from which she had made a good recovery. Several months after leaving the infirmary on that occasion she married, and since then neither she nor her husband has been brought into contact with lead. As there was a complete absence of syphilis and of any local condition to explain the headache and paralysis, it seemed to me that the symptoms might be due to lead. Accordingly I sent a sample of urine to the Chemical Laboratory, Armstrong College, and Professor Bedson reported the presence of lead in the urine. Under treatment by pot. iodide and magnesium sulphate patient got well again. In this patient, after seventeen years of excellent health following an apparent recovery from acute lead poisoning, during which the trials of maternity were many and were well borne and without any fresh infection having taken place, symptoms of an obscure malady develloped, due apparently to a re-solution of lead compounds, which for years had been stored up in the tissues; otherwise how can be explained the presence of lead in the urine? (2) Another case pointing to the slow elimination of the poison is that of a lead mine manager, in whom, although no fresh infection has taken place for four years, and pounds of potassium iodide have been swallowed, lead is present in the urine. The presence of lead in the urine is pretty conclusive evidence of plumbism. It explains the obscure symptoms seen in a few patients who give no history of plumbism or who are known to have suffered from lead poisoning years previously and recovered, and it shows the necessity for the cautious administration of potassium iodide, since this floods the blood with soluble lead salts, which may not only aggravate symptoms, but cause death.

Allusion has been made to the influence of sex in the causation of plumbism. The abolition of female labour in the dangerous processes of a lead factory has already saved thousands of lives. Young women not only become readier victims of lead poisoning than men, they are besides more prone to its serious forms. If pregnant a large number of them miscarry; if term is reached the children are born dead or die soon after birth in convulsions. The only way by which pregnancy can be ended successfully is by getting expecting mothers to retire from lead works during pregnancy. I am of the opinion that no woman should work after the fourth month of pregnancy in any process in which lead is used. Lead not only affects injuriously the pregnant woman herself, but also her offspring, for the poison passes into the placenta, and through it into the fœtus, destroying its life, or causing abortion by the purely ecbolic action of lead upon uterine muscular fibre. Prof. Bedson found lead in the internal organs of stillborn children of female lead workers which I had sent to him; also in several fœtal rabbits, to the mothers of which I had administered lead.

My own experience of the greater predisposition of women than men to plumbism is confirmed by such writers as Constantin Paul in France, whose statistics are most convincing; Sommerfeld, of Berlin; Teleky, of Vienna; and others. Dr. Ignace Kauf*, dealing with type-founders in Austria, male

* "Rapport présenté sur les Intoxications du Plomb. Au Congrès de l'Association Internationale pour la Protection Légale des Travailleurs tenu à Bâle en 1904."

and female, also states that females suffer more from plumbism than males. While from 3.6 to 11.7 per cent. male type-founders become ill, the number of females is from 13.1 to 45 per cent. One-half, or rather 52.4 per cent. of female type-founders when pregnant miscarry, while of those engaged in typographic printing 10 per cent. only miscarry. This shows the varying influence of occupation. Kauf is of the opinion that women working under the same conditions as men suffer more frequently than men and that their illness is of longer duration. Teleky, of Vienna, found in a factory where lead capsules

Fig. 3 (*a*).—Collie Bitch.—Jan. 5.

5 c.c. 10 per cent. $PbN_2O_6$

are made for bottles that of the women who are brushers off and who are therefore exposed to lead 44.1 per cent. miscarry when pregnant, while of the women engaged in other departments in the factory and not exposed to lead only 15.5 per cent. miscarry. It is unfair to attribute all the miscarriages in female lead workers to plumbism—other causes are doubtless in operation, such as the use of drugs and alcohol, syphilis, and the action of the gonococcus, but the operation of these is not limited to one particular section of society or to women employed in one department of an industry.

An early sign of lead poisoning, even sometimes before colic, is anæmia. In chronic cases anæmia is almost always present. It is frequently accompanied by an earthy or subicteric tinge of the skin. In one of my male patients, J. H., aged 40, the red cells were 1,866,660 per c.mm. of blood; in a red lead worker, C. M., who presented no blue line on the gums, but was suffering from paresis of the right wrist with tender supinator longus, and in whose urine Professor Bedson found lead, the red corpuscles numbered 2,916,667 per c.mm. of blood, the white corpuscles 3,902, and the hæmoglobin was 70 per cent.; in an amalgam separator, W. C. C., the red corpuscles were 2,500,000, the white cells 6,000, and hæmoglobin 50 per cent. It would seem, therefore, that in lead poisoning the blood becomes poor in red corpuscles, and with this there is a proportional diminution of the hæmoglobin. It is a simple anæmia, with little or no alteration in the number of white corpuscles, although in one of my female patients, aged 39, a maker-up of blue leads in a lead factory, there was a distinct leucocytosis, the white corpuscles numbering 15,625 and the red 4,316,660 per cmm. of blood. Grawitz drew attention to the presence in plumbism of red corpuscles which had undergone basophile degeneration, with here and there a nucleated red corpuscle. These changes I have occasionally found in the erythrocytes of lead workers in Newcastle, but it is only very few of the corpuscles that are thus affected, and not with the frequency to which other writers have drawn attention. Since these alterations along with poikilocytosis and a varying size of the corpuscles only occur in the older standing cases where anæmia is well developed, and are also met with in other forms of poisoning and debilitating forms of illness unassociated with plumbism, we cannot regard them as pathognomonic of lead poisoning. Dr. Glibert, Principal Medical Inspector to the Belgian Government*, has carried out a series of experiments upon animals to show the effects of lead upon the blood. He finds that some animals are much more rapidly brought under the influence of lead than others, a circumstance which my own experiments confirm. Dr. Glibert finds that when the percentage of hæmoglobin is slowly reduced the resistance of the animal to lead is always greater, and that if the dosage of lead is interrupted the blood tends to recover itself; a circumstance which goes strongly to support the preventive line of treatment I have long advocated, namely, alternative employment of lead workers. While in Glibert's experiments with lead the coloured corpuscles diminished in numbers, this and the fall in the hæmoglobin percentage, although usually running concurrently, were not always observed. A rise in the amount of hæmoglobin is not always accompanied by an increase in the number of red corpuscles. Glibert found not a leucopænia, but rather a slight leucocytosis in several of the guinea-pigs fed with lead.

Fig. 3 (*b*).—Collie Bitch.—Jan. 5.

10 c.c. 10 per cent. $PbN_2O_6$

As it is not my intention to give a detailed symptomatology of lead poisoning, I will set aside colic and the blue line on the gums, merely mentioning that Burton's line occurs at the margin of the gum close to the teeth, and that it is due to the phagocytes in the deeper tissues taking up lead particles in the form of sulphide. Occasionally blue patches the size of a sixpence are seen inside the lips or cheeks

* "Le Saturnisme Expérimental. Extrait des Rapports Annuels de l'Inspection du Travail en 1906."

opposite a decayed tooth. Similar patches may be present in the intestinal tract. As regards colic, Teleky notes a type of hysterical lead colic in young nervous subjects, the peculiar features of which are its long duration and its curability by cold water and electricity.

Loss of vision in lead poisoning occurs under three different conditions. (1) It may be purely toxic and therefore central without any ophthalmoscopic evidence; (2) it may be due to acute neuro-retinitis, with hæmorrhages in severe cases; or (3) associated with and due to vascular changes in the retina dependent upon kidney disease. Very pronounced structural alterations can occur in the retina without albumen in the urine. In a male patient seen in consultation by Dr. Cree of Newcastle and myself, who for four years previously had been little if at all brought into contact with lead, and whose appearance was that of a healthy man, there had occurred a gradual loss of sight. The complaint amounted to an almost complete loss of vision in the lower field of each eye. The loss of vision had taken place without headache, but it had been preceded by colicky pains, which had continued off and on for a few months. There was no blue line on the gums and no paresis or paralysis of muscle. On examining the eyes, Dr. Cree found the right disc extremely pale, except the part internal to the main vessels; the outline perfectly sharp, arteries distinctly narrowed, the veins normal—not tortuous, and no evidence of exudation. In the left eye part of the disc internal to the main vessels was paler than that of the right eye, the arteries seemed narrower in outline and were sharp. In both eyes the vessels encroached upon the macula more than normally. A small cluster of minute hæmorrhages was also observed in the left retina below the disc. By a process of exclusion the various ophthalmologists and physicians who saw this patient came to regard the optic neuritis with commencing atrophy as the consequence of lead. His urine was found to contain lead.

In other lectures I have drawn attention to an irregularity of the pupils, inequality of the radial pulses, unequal severity of pain on pressing one-half of the abdomen compared with the other, and with this pretty severe pain on pressing the vagi in their course in the neck, always worse on the same side as the more painful half of the abdomen. These are frequently to be met with in cases of lead colic, where the pain lingers for several days. One pupil is larger than the other, the tension of one radial artery is higher than the other. As a rule the smaller pupil and the more tense radial artery are on the same side as the pain in the abdomen on firm pressure. While this irregularity of the pupils is observed in the early stages of lead poisoning, and is indirectly the result of the colic, it may also be met with in the more chronic forms in which, in addition to paralysis or paresis of the muscles of the hands and arms and drooping eyelids, there are tremors of the facial muscles and of the protruded tongue, a hilarity of manner and a slowness of speech which recall the saturnine pseudo-general paralysis of French authors, and which under treatment is capable of considerable improvement. Convulsions may occur in the subjects of lead paralysis, whose urine contains no albumen. In a locomotive engine painter, aged 28, the illness began with convulsions followed by loss of consciousness for 3 days. When the coma passed away the right arm and leg were found to be paralysed, and this lasted 3 weeks. There was no albumin in the urine; the amount of urea passed daily was only 130 grains. The presence of a blue line on the gums suggested the diagnosis, which the detection of lead in the urine confirmed. There was marked anæmia, only 2,500,000 red corpuscles per c.mm. of blood and 35 per cent. of hæmoglobin, and yet, notwithstanding this marked alteration of the blood, none of the red corpuscles exhibited basophile degeneration.

With the common affection known as "wrist-drop" in lead poisoning we are all more or less familiar. Although known to ancient writers, it was Boerhaave who in 1656 reported the occurrence of paralysis in workmen handling lead. Since then of the many contributors to our knowledge of the subject it is to Tancquerel des Planches and Duchenne, of Boulogne, that we are mainly indebted. Making use of electricity, Duchenne studied the seat of the paralysis, showed the order in which the muscles became affected, drew attention to paralysis of the short abductors of the thumb, also to a generalised form of saturnine paralysis, and indicated how this might be distinguished from subacute anterior poliomyelitis. Certain types of paralysis have been described. In wrist-drop the common extensors of fingers, and the extensors of forefinger, phalanges of thumb and wrist are affected. The supinator longus, although supplied by the same nerve, escapes. It was Remak who first described in detail the "upper arm" type in which the deltoid, biceps, brachialis anticus and supinator longus are affected, also the "leg" type in which the peroneal muscles and the common and proper extensors of the big toe are involved. In what is known as the Aran-Duchenne type, first described by Mœbius, the small muscles of the hands become affected so as to simulate progressive muscular atrophy. To these may be added the generalised form of paralysis already mentioned, in which the patient lies on his back, unable to move arms, legs, and trunk, and in which there is danger to life from implication of the respiratory muscles. There are also irregular forms of paralysis. The levator palpebræ may be affected so that the eyelid droops, the muscles of the eyeball may not act in concert, or the rectus abdominis may be so affected that the patient cannot sit.

Although the loss of power frequently attacks groups of muscles which are more or less correlated in their action, yet, as Gowers has shown, different parts of a muscle may be affected in different degrees. Some fingers are more paralysed than others, and in the "upper arm" type one part of the deltoid may be weaker than another. Sooner or later the electrical reactions observed in nerve degeneration and known as loss of Faradic response occurs.

(*To be continued.*)

## Figures and Tables

**Fig. 1. f1:**
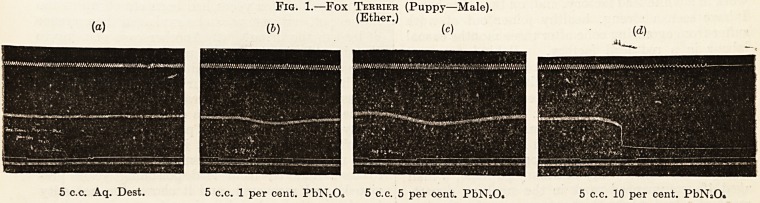


**Fig. 2. f2:**
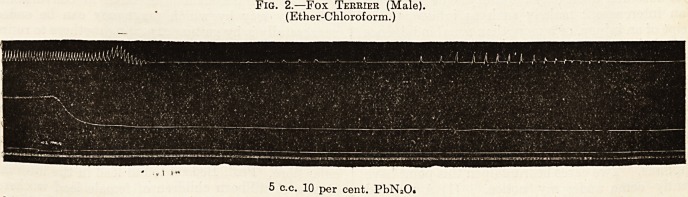


**Fig. 3 (a). f3:**
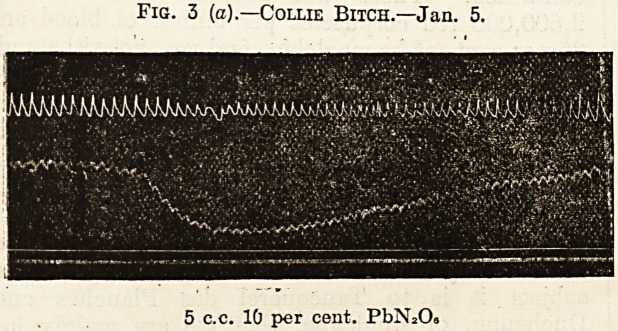


**Fig. 3 (b). f4:**